# Decision making for early surgical technology adoption into Canada’s healthcare system: a scoping review of the decision-making criteria, challenges, and opportunities

**DOI:** 10.1017/S0266462323000363

**Published:** 2023-06-19

**Authors:** Haitham Shoman, Michael Tanzer

**Affiliations:** 1 Vanier Scholar, Institute of Health Policy and Services Research, Canadian Institutes of Health Research, Ottawa, ON, Canada; 2 Department of Surgical and Interventional Sciences (Experimental Surgery), Faculty of Medicine, Montreal General Hospital, McGill University, Montreal, QC, Canada; 3 Division of Orthopaedic Surgery, McGill University, Montreal, QC, Canada

**Keywords:** decision, adoption, surgical technology, healthcare system, Canada

## Abstract

**Objectives:**

In 2020, Canada spent 12.9 percent of its GDP on healthcare, of which 3 percent was on medical devices. Early adoption of innovative surgical devices is mostly driven by physicians and delaying adoption can deprive patients of important medical treatments. This study aimed to identify the criteria in Canada used to decide on the adoption of a surgical device and identify challenges and opportunities.

**Methods:**

This scoping review was guided by the Joanna Briggs Institute Manual for Evidence Synthesis and PRISMA-ScR reporting guidelines. The search strategy included Canada’s provinces, different surgical fields, and adoption. Embase, Medline, and provincial databases were searched. Grey literature was also searched. Data were analyzed by reporting the criteria that were used for technology adoption. Finally, a thematic analysis by subthematic categorization was conducted to arrange the criteria found.

**Results:**

Overall, 155 studies were found. Seven were hospital-specific studies and 148 studies were from four provinces with publicly available Web sites for technology assessment committees (Alberta, British Columbia, Ontario, and Quebec). Seven main themes of criteria were identified: economic, hospital-specific, technology-specific, patients/public, clinical outcomes, policies and procedures, and physician specific. However, standardization and specific weighted criteria for decision making in the early adoption stage of novel technologies are lacking in Canada.

**Conclusions:**

Specific criteria for decision making in the early adoption stage of novel surgical technologies are lacking. These criteria need to be identified, standardized, and applied in order to provide innovative, and the most effective healthcare to Canadians.

## Introduction

Countries have developed healthcare systems in order to ensure people have access to healthcare in a coordinated fashion and ensure the wellness of their nations. The World Health Organization considers health systems as all the organizations, people, and actions that have a primary intent to promote, restore or maintain health including efforts to affect the determinants of health and more directed health-improving tasks (1). Healthcare systems are defined by three main dimensions financing, service provision, and regulation (2). There are four main types of healthcare systems: the Bismarck model, the Beveridge model, the National Health Insurance (NHI), and the out-of-pocket model (2;3). Canada primarily uses the NHI model where the healthcare system is funded directly by income tax deductions and the facilities are owned and operated by the government (2–4), The Canada Health Act, 1984, was developed to ensure eligible residents have universal access to healthcare services (4). Delivery of services is determined by provinces and territories that pool funds into general revenue and the federal government contributes to the revenue pools as per the Health Transfer Agreement (4). Private health insurance can be purchased through employers to cover medical services not covered by the Act (4).

It was estimated that Canada spent approximately CA$ 305 billion on healthcare in 2020, representing 12.9 percent of GDP with an average of CA$ 7,507 per capita. This is above the Organization for Economic Co-operation and Development (OECD) average of CA$ 5,502 per capita (5). The amount spent on medical devices and technologies in Canada is 3–5 percent of the healthcare expenditure, although these estimates are not systematically tracked (4). OECD-developed countries are always looking to improve their healthcare systems and are considered early adopters of new technologies that benefit patients.

Surgery is a highly technical specialty that commonly uses advanced devices and technologies to treat patients. The purchase and adoption of these technologies can occur at any time in the technology adoption life cycle from the innovators to the early adopters, to the early majority, to the late majority, and finally to the laggards (6). The initial decision to adopt surgical technology is by the surgeon, who is the primary user of the device. In the early adoption stage and where there is lack of well-established criteria for decision making, surgeons can decide to adopt technologies based on factors such as (i) surgeon’s preference, (ii) beliefs about the benefit of the technology for their patients, (iii) presentations from conferences, and (iv) information from marketing and sales teams. (7).

Innovation take-up is a dynamic process involving multiple formal/informal decisions by a multitude of interactive factors. In Canada, technology purchase is mainly done through regional health authorities or hospitals via global budgets provided by the provincial health ministries (4). Some provinces tend to use health technology assessments (HTAs) for devices or drugs, but it is unclear at which stage this assessment is conducted. Surgical devices and technologies are one of the most expensive expenditures of the procurement process. Hospitals commonly create technology assessment committees that act as the gatekeepers for the adoption of these new technologies by assessing their value-added benefit (7). However, in the early adoption stage, clinical outcomes of the technology are limited and of short duration, making the assessment of value difficult, if not impossible. As there is limited information on clinical outcomes on surgical technologies at the early adoption stage, which is considered within the exploration stage (stage 2b) of the IDEAL framework, informed criteria for decision making, mentoring, and learning curve evaluation would be considered important (8).

Understanding the role of provincial and local technology assessment committees and the criteria for decision making will help surgeons better recognize the opportunities and requirements to influence the early adoption of innovative technology for the surgical care of their patients (7). The aim of this study is to identify the criteria used by surgeons, hospitals, and provincial bodies and characterize the decision-making process for the adoption of new innovative surgical technology in the Canadian healthcare system. The study will also explore the current challenges and opportunities in the Canadian healthcare system to adopt new technologies to highlight opportunities in other healthcare systems.

## Methods

The methodology for the study was conducted following the Joanna Briggs Institute (JBI) Manual for Evidence Synthesis (9). This study was also reported using the Preferred Reporting Items for Systematic Reviews and Meta-analysis for Scoping Reviews statement guidelines and flowchart (PRISMA-ScR) (10).

### Search strategy

A literature review was conducted using MEDLINE and EMBASE databases, and Google Scholar searching for grey literature. A medical librarian has been consulted for assistance with the keywords and literature search. Provincial HTA Web sites in Canada were also searched along with federal HTA agencies including the Canadian Agency for Drugs and Technologies in Health (CADTH). Search terms were developed to identify articles for the study, and they included the ten provinces and three territories in Canada, all surgical fields, decision making, opportunities, challenges, adoption, innovators, and health technologies. Medical subject headings (MeSH terms) used were “surgical procedure,” “decision making,” “surgical technologies,” “Canada.” All terms were combined using Boolean terms “And”/“Or.” The search terms used are found in Supplementary Table 1.

### Study selection and inclusion and exclusion criteria

The study selection and screening were conducted by two independent reviewers and there were no disagreements. This review only included articles published from inception until December 2021. Articles included were observational studies, randomized trials, HTA’s, case studies, and series. The study included articles focused on the decision-making process for early new surgical technology adoption into clinical practice, articles published in English and French, articles that focus only on the Canadian healthcare system and its thirteen provinces and territories and articles that explore the strengths and weaknesses in the Canadian system for technology adoption. The articles that focused on the decision-making process include whether these were decisions already made to adopt a technology, or decisions yet to be made by physicians. All hospital-based and province-based studies were considered and screened for eligibility according to the inclusion criteria and were then referred for full-text assessment. Articles outside Canada, in languages other than English and French which did not include adoption of technologies were excluded from this study.

### Data extraction

Articles found were imported into Endnote X9 reference manager software where duplicates were removed and the filtration process for all studies took place. There were no disagreements between authors. The data was then extracted into a spreadsheet created in Microsoft Excel (Table 1). The data extracted included information based on the author and year the article was published, the level of evidence and study type, the geographic location, surgical specialty, the surgical device (technology), decision-making framework and criteria, challenges, opportunities, and general applicability. Data were also extracted from provincial Web sites identifying the criteria used and the responsible HTA agency (Table 2).

**Table 1.** Data extraction from database search

Author, year (location)	Level of evidence (study type)	Surgical specialty (technology)	Stage on the adoption curve*/*decision process framework	Decision-making criteria and process/opinion	Challenges	Opportunities	General applicability
1. Goeree, 2009 (Ontario)	NA (HTA policy review)	Coronary Artery Disease (Drug eluting stents)		Cost, social, ethical values, legal issues, feasibility of implementation. Quality, safety, efficacy, effectiveness, value for money	Even after careful consideration of the evidence from well-conducted HTAs, decision makers may still have residual uncertainty around a number of issues	Provide a useful evidence-based framework for decision making. Primary data collection is often considered a supplement to evidence available from traditional HTAs.	The HTA process in Ontario represents an interesting adaptation to the traditional HTA approach because primary data collection is used to supplement the HTA, and the iterative evidence-based PRUFE framework, through the use of VOI analysis, is used to help determine research feasibility and data collection needs within studies
2. Borowski, 2007 (Alberta)	NA (HTA review)	Several (Several)	Alberta Heritage Foundation for Medical Research	Social/demographic, Technological, Environmental, Economic, Political (STEP), Legislative and Ethical considerations	Some HTAs did not include the STEP framework that is now in use. The original framework included five elements: population health impact, technological effectiveness, economic evaluation, implementation issues, and policy analysis. We found that some elements could be combined or addressed more fully at other points in the process	It could enhance the prominence of evidence, while enabling decision makers to do the balancing required for policy formulation	The Alberta Health Technologies Decision Process has greater chance for success in informing policy, because it recognizes that policy and decision makers in government prefer to incorporate or balance other factors and information beyond hard evidence when making decisions
3. Danjoux, 2007 (Ontario)	4 (Case study)	Abdominal aortic aneurysm (Endovascular aneurysm repair)	Accountability for Reasonableness	Relevance, publicity, appeals, enforcement – Medical individualistic perspective	Not generalizable for smaller hospitals, small sample size	Hospitals should develop a structure for deliberating the reasons for adopting a surgical innovation that involve a wide range of stakeholders. Broader input should be sought from individuals involved with the procedure and those at “arms length” who may not be directly invested in the innovation. Hospitals should establish a formal appeals mechanism for addressing challenges to the decisions being made.	NA
4. Lehoux, 2012 (Canada)	4 (Case study)	Several (Several)	Socio-technical dimensions and features (prevention, efficiency, sense of security, real-time feedback, ease of use, flexibility)	Clinical (Impact on clinical activities and outcomes), Technical (Technical assets and comparison with technological alternatives), Structural (Impact on work processes and health care structures), human (Response to clinicians’ and patients’ values, expectations and constraints)	Lack of ethical appeals, evidence, lack of universal strategies	Manufacturers’ Web sites can bolster physician and patient expectations that can then be easily used to put pressure on third-party payers	Our study also showed that the valuable socio-technical goals and features that manufacturers invoke are, at first glance, in tune with the challenges of modern health care systems. However, the reference to these values is clearly more rhetorical than demonstrative
5. Poulin, 2012 (Alberta)	NA (Review)	Several (Several)	Based on HTA Calgary Health Region program	Health gain (efficacy, population health, standard of care), Service delivery (safety, training, access, service coordination), Sustainability (long term), Strategic fit (good alignment with local values), Innovation (Knowledge and research), Financial (cost, economic analysis)	HTA program lacks patient and public input.	Local HTA Program is positioned to help bridge the gap between evidence and practice, by providing a way to incorporate global evidence with local relevance and involving surgeons themselves. Hospital-based HTA using local data can fill gaps in the published evidence and also improve the generalizability of evidence to the local setting. Hospitals should maintain easy access databases.	We believe that the program is generalizable to other health care organizations that require integration of local contextual information with research evidence as provided in external HTA reports. The Program has sufficient versatility to be adapted to a wide variety of regional health authorities.
6. Sharma, 2006 (Ontario)	4 (Case study)	General surgery (Advanced laparoscopic surgery)	Accountability for reasonableness	Relevance, publicity, appeals, enforcement	Recall bias, social desirability bias	Ways to improve the fairness and legitimacy of decision making, including (i) publicizing the process and results of decisions about the adoption of new surgical technologies, (ii) clarifying and publicizing the role of the hospital board in providing a structure for appeals of decisions, and (iii) impaneling a group of stakeholders, with public representation, to oversee the decision-making process. Our findings will help physicians and health care administrators improve the decision-making processes for innovative surgical technologies	Observations based on one community hospital and not generalizable
7. Urquhart, 2014 (Nova Scotia)	4 (Case study)	Several (Several)	Organizational framework of innovation implementation	Management support, financial resource availability, implementation policies and practices, implementation climate, innovation champions, and innovation-values fit	A number of key informants stated it was difficult to remember what happened during the implementation period. Therefore, the data are subject to issues of recall bias.	The findings revealed that positive relationships can counterbalance many negative contextual factors – thus, the early engagement of key stakeholders across multiple levels of healthcare organizations and systems may be fundamental to implementation efforts and to supporting the consistent and committed use of an innovation. The findings also demonstrate the importance of a multilevel contextual analysis to gaining both breadth and depth to our understanding of innovation implementation and use in health care.	Given that the structure and socio-political context of healthcare systems vary, this may limit the applicability of findings to other jurisdictions. Nonetheless, healthcare systems generally have a number of defining features, including a wide range and diversity of stakeholders, complex governance and resourcing arrangements, and high degrees of professional autonomy of many of its staff, which should increase the applicability of these findings in other health systems

HTA, health technology assessment; NA, not applicable.

**Table 2.** Data extraction from provincial Web sites

Item	Alberta	British Columbia	Ontario	Quebec
No. of studies	32	22	302	415
Surgery-related studies	11	7	90	40
Agency name	AHT – DP (Alberta Health Technologies Decision Process)	HTR (Health Technology Review)	HQO (Health Quality Ontario) Evidence, Developments and Standards Division	INESSS (Institut National d’Excellence en Santé et en Services Sociaux)
HTA method	Systematic review – Economic evaluation – Budget Impact Analysis			
Criteria	Social and system demographics (incidence/prevalence – service delivery capacity)	Social and system demographics (disease burden – population impact – training and credentialing required)	Social and system demographics (disease burden – need)	
	Clinical effectiveness (Health and non-health effects)	Clinical effectiveness (Health and non-health effects – quantity and quality of life)	Clinical effectiveness (Safety – effectiveness)	Clinical effectiveness (Safety – effectiveness)
	Political and public policy considerations	Political and public policy considerations (access – environmental impact – prevention – risk to implementation – impact on marginalized/disadvantaged patients)	Political and public policy considerations (societal and ethical values)	
	Costs	Costs	Costs	Costs
			Feasibility of adoption into health system (economic – organizational)	

HTA, health technology assessment.

### Data synthesis and analysis

Articles found were grouped into hospital-based and province-based studies. Criteria that were used by physicians in the decision-making process for technology adoption were collected and reported. The frequency of reporting of each criterion was also collected. The criteria were then grouped and classified based on a thematic categorization of all the criteria and guidance sought from previous studies (11;12). Finally, the surgical technologies identified in the studies were grouped into surgical fields along with when they were adopted.

## Results

**Figure 1.**
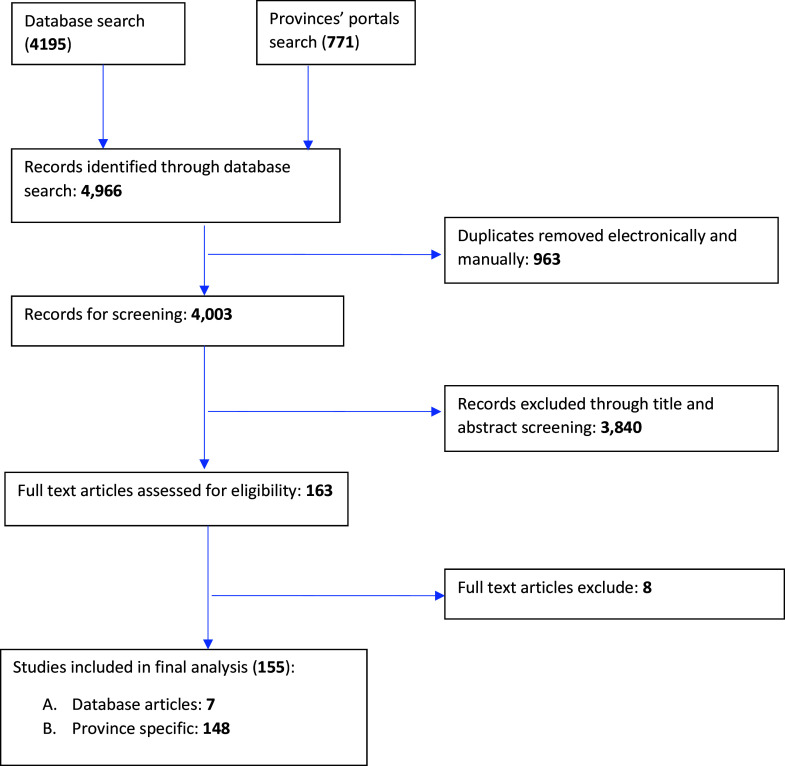
PRISMA (scoping review) flowchart.

**Figure 2.**
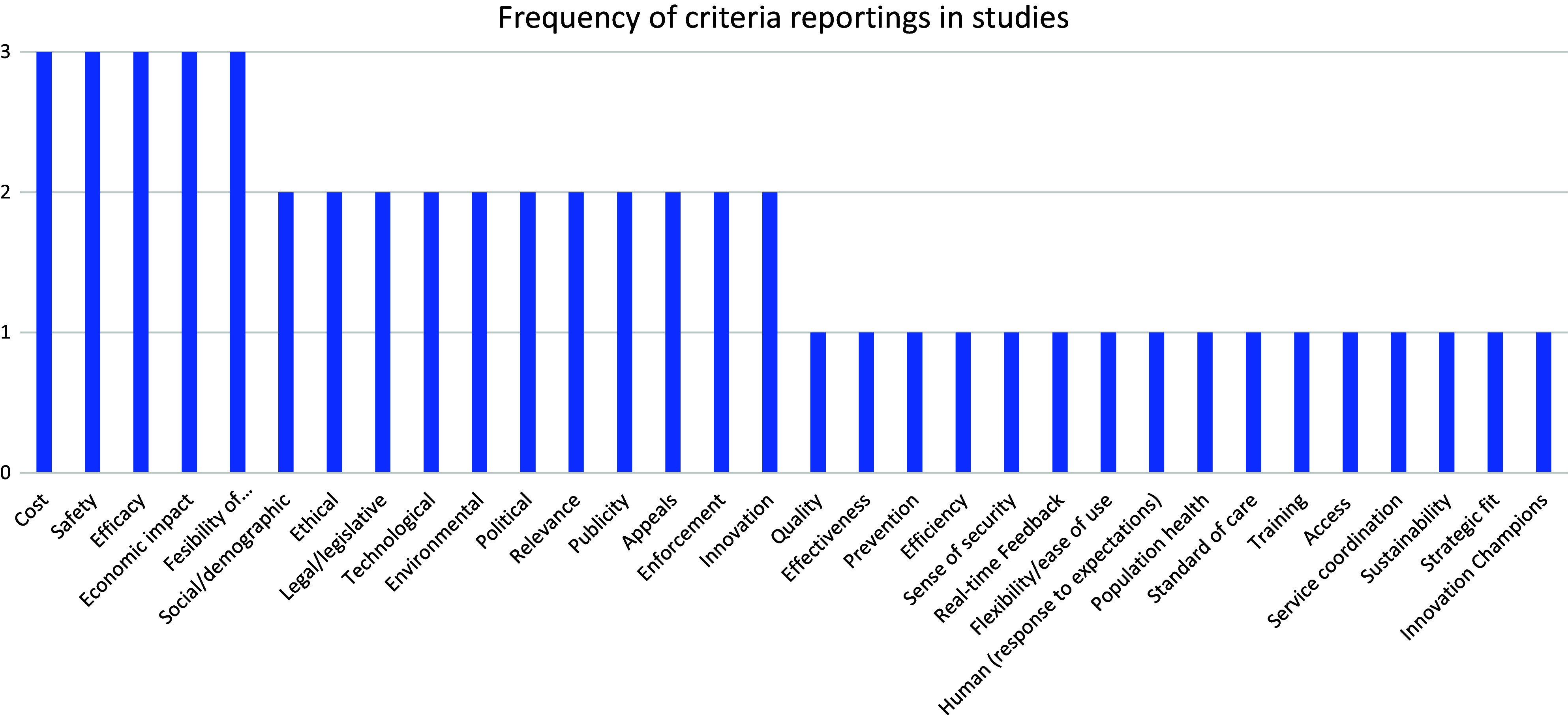
Frequency of criteria reporting from studies.

The search strategy for this study yielded a total of 4,966 articles (4,195 from the database search and were hospital-based; and 771 from provincial Web sites). After duplicates were removed and screening was done, the searches identified 155 articles that met the inclusion criteria (Figure 1). Of these, 148 were HTA reports from provincial Web sites, four were case studies and three were policy review articles. A total of ninety-three articles were from Ontario, forty were from Quebec, thirteen were from Alberta, seven were from British Columbia, one was from Nova Scotia and one was a national study. The technology assessed included surgical devices for cardiothoracic surgery, general surgery, obstetrics and gynecology, orthopedics, and ophthalmology. None of the articles indicated in which stage of the technology adoption life cycle the technology was in at the time of its review. Figure 2 shows all criteria and subcriteria found from the search strategy.

### Criteria elicitation – HTA reports from provinces

All 148 provincial HTA reports used the same methodology which included a systematic review, an economic evaluation, and a budget impact analysis of the technology. Provinces used a set of criteria that were determined by each province and were only standardized across the HTA reports that they used (13). Table 2 shows a summary of the information gathered from the provincial Web sites. The price of the technology and its clinical effectiveness (safety and effectiveness) were the most important criteria used in the decision-making process in all the provinces (2–6). In three provinces, political and public policy considerations, as well as social and system demographics (incidence and prevalence of the condition) were used to guide their decisions and they were considered additional criteria (13–17). The political and public policy considerations include access to the technology, environmental impact, prevention of diseases, risk of implementing the technology, and impact on marginalized/disadvantaged patients. One of the provinces also used societal and ethical values in considering which technologies to adopt along with the feasibility of adoption into the healthcare system (13–17). Cost, safety, efficacy, economic impact, and feasibility of implementation of the technology were the most frequently reported criteria across all studies. This is in line with the provincial priorities on what guides them to adopt new technologies into their hospitals.

### Thematic groupings and criteria elicitation (from the seven articles)

Seven articles from the database search identified the priority criteria that surgeons use in their decision-making process to adopt a new surgical device (18–24). All the criteria were gathered systematically by the authors using structured methodologies from the JBI Manual for Evidence Synthesis (9). Overall, thirty-three criteria were identified as influencing surgeons, and other healthcare professionals, in adopting a new technology. The criteria were extracted from Table 1. The methodologies used include using different qualitative frameworks with questionnaires designed to ask surgeons what is considered important in their decision-making process. These frameworks are the Alberta Heritage Foundation for Medical Research Framework, the Accountability for Reasonableness, the Socio-technical dimensions and features, the Calgary Health Region HTA, and the Organizational Framework of Innovation Implementation.

The thirty-three criteria had recurrent themes and could be categorized by thematic categorization into seven distinct groups of criteria (Table 3). Group 1 includes all the criteria that relate to the economics of the technology. Group 2 includes the hospital-specific criteria and refers to how this device fits into the hospital’s ecosystem, integration, and workflow. Group 3 includes the technology-specific criteria and refers to features that define the device and its specifications. Group 4 is the relevance to patients and the public and these criteria refer to the usability of the technology/device to the overall population and their feedback. The Group 5 criteria are related to clinical outcomes from the clinician’s perspective. Group 6 is policies and procedures criteria and refers to regulations in the country/hospital that facilitate integration and ease of usability of the technology. Finally, Group 7 are criteria that are physician-specific and refers to how the physician interacts with the technology. It is worth noting that there was no weighting of any of the thirty-three criteria in considering which is more important among the studies.

**Table 3.** Categorization of criteria for decision making

Category	Criteria
1. Economic	Cost of technology Economic impact
2. Hospital specific	Feasibility of implementation Structural/management support Strategic fit Relevance Standards of care Service coordination
3. Technology specific	Technology simplicity Innovation Quality Real-time feedback Efficiency
4. Patients/public	Population health impact Human responses (patient experience) Publicity and awareness of technology Access to technology Social/demographic
5. Clinical outcomes	Safety Efficacy Effectiveness Adverse events and prevention
6. Policies and procedures	Ethical Legislative Environmental Sustainability Political Appeals Enforcement
7. Physician specific	Sense of security Flexibility of usage Innovation champions Training

### Challenges

Three of the studies identified challenges with the criteria used by surgeons to adopt novel technology. First, there is expressed uncertainty about whether or not these criteria were generalizable for all technologies in all surgical specialties (20). Second, there was potential bias in the surgeon’s criteria, thereby limiting its applicability as these criteria were prioritized by physicians in large hospitals, and may not be generalizable nor applicable to smaller hospitals with smaller budgets and limited access (19). Third, Canada’s healthcare system presently lacks a universal strategic system with a guide on how to adopt new technologies (21). Furthermore, provincial HTAs only assess technology based on cost-effectiveness, budget impact, and clinical outcomes.” Two of the studies found that there might be a recall bias where some physicians could not recall the last time, they decided on how or why should a new technology be adopted (23;24).

### Opportunities

All seven studies, including the two studies that utilized provincial HTA criteria, identified specific opportunities that could help improve the Canadian healthcare system in procuring new technologies at the early adoption stage. These opportunities addressed the process at both the provincial and hospital level. First, surgeons with well-defined criteria to adopt a new technology would help in providing a useful evidence-based framework for decision making. This primary data collection is considered a supplement to the evidence available for formal HTA reports (20). Second, criteria gathered from surgeons would help enhance the strength and availability of evidence while enabling decision making to balance what is needed for policy formulation (18). Third, such criteria collected would help in triggering hospitals to better develop a structure that would involve wider stakeholders for more input and prompt the development of a comprehensive appeals mechanism for addressing challenges to decisions made (19). Fourth, such criteria would help manufacturers create Web sites for these products that would bolster the surgeons’ expectations and needs by answering their questions based on the criteria already gathered a priori (21). This would help create a more transparent platform for surgeons to make more informed decisions. Fifth, HTA programs available locally and nationally help bridge gaps where evidence is lacking to support surgeons’ knowledge of a new technology for more informed decisions (22). Sixth, public representation along with physician’s expertise to ensure public and patient insights are taken into consideration (23). Finally, the adoption of technologies should involve internal multilevel stakeholders’ such as administrators, other health professionals and hospital decision makers early in the process to facilitate uptake and adoption as there are regulations that can either prompt or hinder adoption apart from surgeons’ needs (24).

### Applicability

The views on the applicability of using these criteria amongst all provinces and hospitals differed between authors. In two studies, it was contended that the observations in one hospital and community might not be generalizable because of the diverse structure and socio-political context of healthcare systems in different jurisdictions (23;24). In addition, this is further confounded by a wide range and diversity of stakeholders, complex governance structures, resource arrangements, and high degrees of professional autonomies (24). Two of the studies felt that the inclusion of the surgeons in the decision-making process made the adoption assessment more applicable and universal. Goeree et al. (20) speculated that when physicians’ criteria for adoption are supplemented by HTA reports outcomes, it could help create more informed decisions that would be applicable to different settings. In addition, applicability to other systems and feasibility of such criteria could be possible when decisions include several stakeholders, especially from the government, so that there is a balance from a multitude of factors including the regulatory environment (18).

## Discussion

The aim of this study was to identify the criteria used to determine the decision making for new technology adoption. Thirty-three criteria were identified and grouped into seven categories named: economic, hospital-specific, technology-specific, patient-specific, clinical outcomes, policies and procedures, and finally physician-specific. In the Canadian healthcare system, there is no standardization of decision-making criteria in technology adoption. Although there is some overlap between the criteria felt to be important by surgeons and the provincial/hospital committees, the provincial and hospital committees focus primarily on the cost of the technology and its clinical effectiveness. This limits the opportunity for the adoption of innovative technology in the early adoption stage as there is only limited outcomes information available from the innovators. However, this study identified multiple opportunities to help improve the Canadian healthcare system in procuring new technologies at the early adoption stage.

### Prioritization criteria in Canada

CADTH, created in 1989, is the main agency that coordinates an approach for all HTAs to produce evidence-informed recommendations that will assist decision makers and benefit patients (25). CADTH has identified priority-setting criteria for new technology assessment and adoption based on the EUR-ASSESS project and then conducted a multiple-criteria decision making (MCDA) to weigh the criteria and identify priorities based on the weights after consultation with selected committees (12;26). The assessment was based on all new technologies and drugs; and the selected committee members were mainly representatives from federal, provincial, and territorial publicly funded drug plans and pharmacists working for the ministries of health (12). No surgeons/physicians were included in these committees who are considered the ultimate users of these technologies. The CADTH study revealed that the clinical impact of technologies carries the highest weight for decision makers, followed by (in descending priority order): the burden of disease, the economic impact, budget impact, availability of evidence, and alternatives for the technology (12). The process for device use in Canada requires that the product receive Health Protection Branch of Canada (HPB) approval or HPB approval for a batch release to conduct a clinical trial.

### The “value” in decisions

Value is broadly known as the ratio of quality to cost, but this varies among healthcare stakeholders (7). The global landscape view on value has challenged leaders to explore new models to engage clinicians for shared risk and rewarding successful adoption for improved patient outcomes. Such value committees are growing today more than ever due to the pressing global challenges from natural threats, industrialization, globalization, economic pressures, and changing patients’ needs. In Canada, there has not been a comprehensive study that explores the prioritization criteria for decision making for surgical technology early adoption from the surgeon’s perspective. As well, the criteria presently used for technology adoption are most applicable during and after the early majority stage, when clinical outcomes and longer-term follow-up become available. They do not specifically address the criteria to adopt technology in the early adoption phase, when there is limited outcome data from the innovators, that only make up 2.5 percent of the users. Involving surgeons, the end-users, and making them part of such decisions, or even developing a criteria framework based on surgeon’s decisions in the evaluation of new technologies, would be a more tailored approach that would eventually benefit patients (7). The IDEAL framework has proposed the assessment of surgical innovation based on a five-stage description of the surgical development process; innovation, development, exploration, assessment, and long-term study (8). Early adopters can be involved in the development and improvement of the technology but are primarily involved very early in the exploration phase. This phase uses early and limited prospective and collaborative cohort studies to focus on the learning curve, the indications for the innovation, and its quality. These criteria are some of the assessment tools identified in our review, specifically in the categories of clinical outcomes, physician specific and technology specific. This can prompt the development of controlled trials in the exploration stage where the learning curve can affect surgeons’ involvement in these studies as they can identify relevant outcome measures (8). These measures would be crucial for research databases and trials and would include technical, clinical and patient-reported outcomes to help provide further information about the technologies used and guide other surgeons for making informed decisions (8).

The limitations of this study would help prompt further research in criteria prioritization. There was a lack of any quantitative metrics for criteria weighting based on the results we found. This makes it challenging to identify which criteria are considered a priority over another. Another limitation is that the results found may not represent all of Canada as most of the results found were attributed to only four provinces’ HTA reports. Most of the studies and reports did not factor in the surgeon’s perspective and priorities in technology adoption. In addition, many of the studies in the literature are older and it is unclear how well, or if they are reflective of current practice. However, it does indicate the need for further studies that explore the changing dynamics of health systems and patients’ needs. More research is needed to challenge and validate the criteria using quantitative metrics to weight and prioritize them for guiding surgeons with informed decision making for the early adoption of new surgical technologies in the Canadian healthcare system. As well, the relative weight of each criterion may vary by geographic region, healthcare system, and hospital.

## Conclusion

The economic and clinical impact of new technologies is the two most important criteria for technology adoption in healthcare in Canada. The findings of the scoping review have also highlighted some of the deficiencies in the present literature. Value assessment committees should include surgeons in the decision-making process and more research is needed for a comprehensive study that would explore the surgeon’s perspective in criteria prioritization for technology adoption. Further studies are needed from other provinces to help have a representative set of weighted criteria that would be applicable to the entire country. Specific criteria for decision making in the early adoption stage of novel technologies are lacking. These criteria need to be identified, standardized, and applied in order to provide innovative, and the most effective healthcare to Canadians.

## Supplementary material

For supplementary material accompanying this paper visit https://doi.org/10.1017/S0266462323000363. click here to view supplementary material
