# Supplementary material for: Decision making for early surgical technology adoption into Canada’s healthcare system: a scoping review of the decision-making criteria, challenges, and opportunities
Source: Int J Technol Assess Health Care. 2023 Jun 19;39(1):e41. doi: 10.1017/S0266462323000363 (PMC11569962; doi:10.1017/S0266462323000363)
Supplement: Supplementary file 1 [file S0266462323000363sup001.docx]

**Table 1: Search strategy using MEDLINE database.**

| **Count** | **Search terms** | **EMBASE** | **MEDLINE** |
| --- | --- | --- | --- |
| 1 | **("decision making" OR opportunit* OR challenge OR "health technolog* assessment" OR "adoption curve" OR adopt* OR innovators OR "early adopters"**).mp. [mp=title, abstract, original title, name of substance word, subject heading word, floating sub-heading word, keyword heading word, organism supplementary concept word, protocol supplementary concept word, rare disease supplementary concept word, unique identifier, synonyms] | 1652285 | 1153633 |
| 2 | **(Surgery OR "surgical intervention" OR surgical OR neurosurgery OR orthop?dics OR urology**).mp. [mp=title, abstract, original title, name of substance word, subject heading word, floating sub-heading word, keyword heading word, organism supplementary concept word, protocol supplementary concept word, rare disease supplementary concept word, unique identifier, synonyms] | 4663610 | 3281573 |
| 3 | **("Canadian health system" OR "Canadian healthcare system" OR "Canada health system" OR "Canada healthcare system" OR Canada OR ontario OR quebec OR alberta OR "british columbia" OR manitoba OR Saskatchewan OR yukon OR "New Brunswick" OR "Newfoundland and Labrador" OR Northwest Territories OR "Nova Scotia" OR Nunavut OR "Prince Edward Island**").mp. [mp=title, abstract, original title, name of substance word, subject heading word, floating sub-heading word, keyword heading word, organism supplementary concept word, protocol supplementary concept word, rare disease supplementary concept word, unique identifier, synonyms] | 294214 | 227802 |
| 4 | 1 AND 2 AND 3 | 2617 | 1578 |
